# Successful integration of an automated patient-reported outcome measure within a hospital electronic patient record

**DOI:** 10.1093/rap/rkac065

**Published:** 2022-08-17

**Authors:** Matthew T Neame, David Reilly, Ajmal Puthiyaveetil, Liza McCann, Kamran Mahmood, Beverley Almeida, Clare E Pain, Victoria Furfie, Andrew G Cleary

**Affiliations:** Department of Information Technology, Alder Hey Children's Hospital; Department of Women’s and Children’s Health, University of Liverpool; Department of Information Technology, Alder Hey Children's Hospital; Department Rheumatology, Alder Hey Children's Hospital, Liverpool, UK; Department Rheumatology, Alder Hey Children's Hospital, Liverpool, UK; Department Rheumatology, Alder Hey Children's Hospital, Liverpool, UK; Department Rheumatology, Alder Hey Children's Hospital, Liverpool, UK; Department Rheumatology, Alder Hey Children's Hospital, Liverpool, UK; Department of Information Technology, Alder Hey Children's Hospital; Department Rheumatology, Alder Hey Children's Hospital, Liverpool, UK

**Keywords:** Medical informatics, patient-reported outcome measures, rheumatology, quality improvement, paediatrics

## Abstract

**Objectives:**

The objective of this evaluation was to assess the feasibility of implementing a fully integrated, automated, electronic patient-reported outcome measures (ePROM) system into a hospital electronic patient record (EPR; hospital-based clinical record). Additional objectives included evaluating the effect of the system on patient-reported outcome measures (PROM) completion rates and investigating the acceptability of the ePROM.

**Methods:**

The evaluation was conducted in a rheumatology clinic in a specialist children’s hospital in the UK. Paper-based childhood HAQ PROMs were already used in the clinic, and an EPR was the main hospital information system. The technical feasibility of introducing the ePROM technology was assessed using a case study approach; the effect of the system on PROM completion rates was investigated using a before–after design; and acceptability was assessed using semi-structured questionnaires and a focus group.

**Results:**

An automated and integrated ePROM system was implemented successfully in April 2021. After implementation, ∼500 automated SMS text messaging invitations to complete ePROMs were sent to care-givers each month. PROM completion rates increased from 33 of 100 (33%) to 47 of 65 (72%) after the introduction of the ePROM system (χ^2^ = 11.51; *P* < 0.05). The ePROM system was highly acceptable to patients and clinical staff. Some clinical staff expressed a concern that an electronic system might represent a barrier to care for families with more limited resources.

**Conclusion:**

High levels of automation and integration with existing technology systems seemed to be key contextual factors associated with the successful implementation and adoption of the ePROM intervention in a paediatric rheumatology clinic.

Key messages

- The development of an automated, fully integrated electronic patient-reported outcome measurement (ePROM) system was feasible.
- The ePROM system improved PROM completion and documentation rates.
- The ePROM system was highly acceptable to health professionals and patients.

## Introduction

Patient-reported outcome measures (PROMs) are instruments for assessing health conditions from a patient or caregiver’s perspective. PROMs are most frequently designed as standardized, validated questionnaires, and they can be used to measure the health effects (outcomes) that are of most importance to patients; these might include levels of physical or social functioning, severity of symptoms or general well-being [1].

PROMs have been key to improving the quality of rheumatology research [2] and are also used widely in routine clinical care [1]. In research settings, PROMs can help to identify the treatments that offer the most beneficial effects. In routine clinical care, they can enable health-care workers and patients to track the effects of treatments and can enable more objective audits of the services provided by different health-care organizations [1, 3, 4].

The child HAQ (CHAQ) is a 36-item PROM that has been validated for assessing functional outcomes, general well-being and pain [5]. The rheumatology team at Alder Hey Children’s NHS Foundation Trust, a specialist children’s hospital in the UK, have historically used the CHAQ [6] as a routine standard of care in outpatient clinic consultations.

However, informal interviews with health-care professionals (HCPs) and patient groups highlighted issues that negatively impacted CHAQ completion and documentation rates. These factors included: paper CHAQs not being handed out to all eligible patients at appointments; the need for HCPs to score and document completed assessments manually; lack of access to paper CHAQs outside clinic settings; and a risk of calculation and transcription errors associated with manually scoring and documenting results into the hospital’s electronic patient record (EPR).

An electronic PROM (ePROM) system was identified as having the potential to reduce the impact of these issues. This evaluation was therefore conducted with the following three objectives: to investigate the feasibility of implementing an ePROM system with automated generation of requests, and automated scoring and integration of data into the EPR; to study the effects of using the system on CHAQ completion rates; and to evaluate the acceptability of the system.

## Methods

The methods used included the following: a descriptive case study to investigate the feasibility of implementing the technology; a before–after study to evaluate the effects on CHAQ completion rates; and surveys and focus groups to gather data about the acceptability of the intervention.

An evaluation protocol (Supplementary Data S1, available at *Rheumatology Advances in Practice* online) was registered with the Alder Hey Children’s NHS Foundation Trust Governance and Assurance department. National Health Service (NHS) Health Research Authority guidance [7] indicated that Research Ethics Committee approval and formal written consent were not required because the investigation constituted a service evaluation exercise that used anonymized data. The Standards for Quality Improvement Reporting Excellence (SQUIRE 2.0) guidelines [8] were used to structure this report.

### Feasibility case study

The technical feasibility case study was conducted using data gathered from direct observations, technical specification reports and schemas, meeting notes, internal reports and internal presentations. The case study report was developed using SQUIRE 2.0 [8] and Health Information Technology (HIT) [9] reporting guidelines.

### CHAQ completion rates

The effect on CHAQ completion rates was investigated using a before–after study design. Data were identified from manual reviews of the clinical records of children who attended scheduled rheumatology appointments in a 2-week period before (July 2019) and after (July 2021) the introduction of the ePROM system.

In the before period, clinical records were defined as demonstrating a completed CHAQ if they included any documentation of a CHAQ score. In the after period, the records were identified as demonstrating a completed CHAQ assessment if they included a CHAQ score as structured data against a specific CHAQ score query in the EPR and as an appropriately filed electronic copy of the completed CHAQ questionnaire. Chi-square testing was used to analyse whether differences in completion rate were statistically significant.

### Acceptability of the ePROM intervention

The acceptability of the intervention was evaluated using a patient survey and using a focus group and survey of HCPs.

#### Patient survey

The patient and parent ePROM questionnaire was developed by the evaluation team based on findings from systematic reviews of HIT implementation research [9, 10]. These reviews identified key constructs that are associated with successful HIT implementations, including attitudes and acceptance of health technologies and the accessibility of HIT systems. The questionnaire items were developed through iterative discussion rounds by the evaluation team, and questionnaires were administered to families who were attending outpatient clinics in July 2021 (in paper format to avoid excluding families who did not have access to electronic questionnaires). The data from these questionnaires were analysed using descriptive statistical methods. A copy of the questionnaire is available in Supplementary Table S1, available at *Rheumatology Advances in Practice* online.

#### Focus group with health-care professionals

A focus group was conducted with rheumatology HCPs. The focus group method was selected to encourage dialogue highlighting the experiences of HCPs using the ePROM system and was conducted as part of a scheduled team meeting held online (MS Teams software). Two investigators (M.T.N. and A.G.C.) provided a brief presentation introducing the objectives of the ePROM project and describing the purpose of the focus group (slides presented as Supplementary Fig. S1, available at *Rheumatology Advances in Practice* online). M.T.N. led a guided discussion covering general observations relating to the ePROM system, followed by a directed discussion of its strengths, weaknesses and unintended consequences, and data were collected from recorded meeting minutes and field notes. M.T.N. and A.G.C. then led an inductive analysis, coding and categorizing the data into themes that were described by the HCPs.

##### Survey of HCPs

The technology acceptance model 2 (TAM2) questionnaire [11, 12] was used to assess the acceptability of the ePROM system to HCPs. TAM2 has been validated for use in workplace settings and is designed to measure key constructs that predict usage intentions and the acceptance of workplace information technology systems [13]. The items are measured using seven-point Likert scales. Invitations to complete the questionnaire were sent electronically to 29 members of the rheumatology Multi-Disciplinary Team on two occasions in July 2021. The results of the survey were analysed using descriptive statistical methods.

## Results

### Feasibility case study

#### Evaluation setting and existing health information technology infrastructure

The main clinical information system used in the Trust throughout the study period was an electronic patient record (EPR; Meditech v.6.08, Boston, MA, USA). EPR functions included reviewing and scheduling appointments, documenting consultations, and requesting and reviewing medication and investigations.

Paper-based CHAQ PROM assessments had been used as a part of routine clinical care within the rheumatology department on a long-term basis. Previously, paper questionnaires were provided to patients for completion in the clinic waiting room and were then passed to clinical staff for scoring/transcription during the consultation.

#### Development of the ePROM system

The ePROM system was developed by clinical leads from the Rheumatology team, representatives from the Alder Hey Information Technology team, and an independent technology provider (Aire Logic, Leeds, UK). Consultation with patient groups and literature reviews identified web links within Short Messenger Service (SMS) text messages as an acceptable and accessible approach for contacting families [14]. Literature reviews and consultations with HCPs who used PROMs highlighted automation and integration with existing HIT systems as key factors for improving the chances of implementing the system successfully [15–17].

A data protection impact assessment (DPIA) was completed and presented to the Trust’s Information Governance committee, who approved the project in October 2019.

**Fig. 1:**
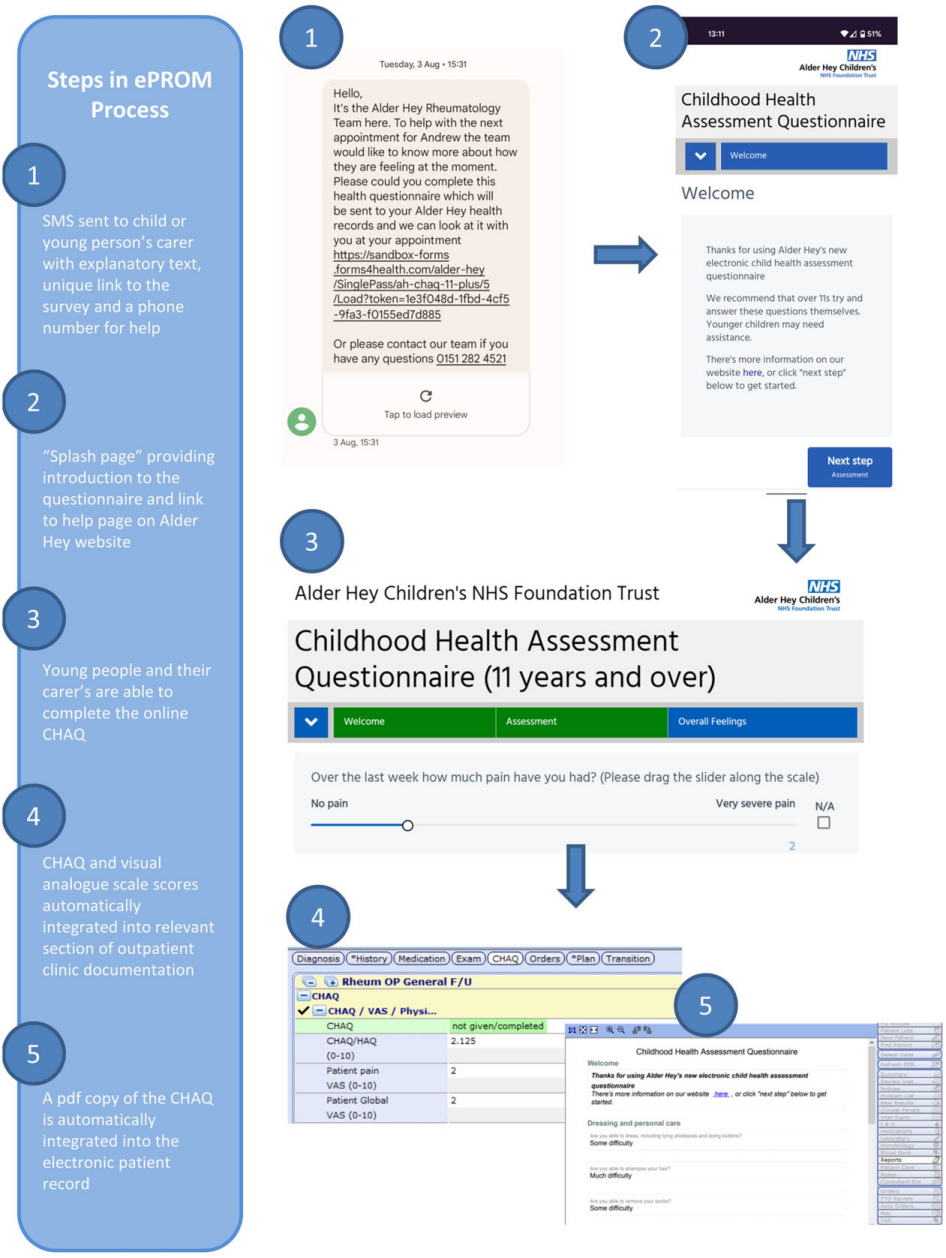
Schema and screenshots illustrating the functions of the electronic patient-reported outcome measures system CHAQ: childhood HAQ; ePROM: electronic patient-reported outcome measures.

The system was developed with the following technical features (see Fig. 1 and Supplementary Fig. S2, available at *Rheumatology Advances in Practice* online, for screenshots of patient and clinician user interfaces):

1. A daily report was generated to identify patients scheduled to attend rheumatology clinics (Meditech EPR reporting module).
2. Patient data were pseudonymized (Aire Glu; Aire Logic) in order to generate a unique web address [uniform resource locator (URL)].
3. The URL link to the online CHAQ assessment was delivered to the relevant mobile telephone number listed in the patient record via an SMS text message (CHAQ assessments provided by Forms4Health software; Aire Logic).
4. Completed CHAQs were re-associated with the relevant patient record (AireGlu; Aire Logic) before being stored against the relevant clinical episode/visit.
5. Numerical CHAQ scores were calculated and visible to clinicians in electronic forms used in the rheumatology outpatient documentation, and pdf versions of the assessments were also stored against the patient visit.

No formal training was provided to either HCPs or families, because the system was designed to be as automated and accessible as possible.

#### Implementation of the ePROM system

The ePROM system was launched in March 2021, with ∼500 invitations to complete ePROMs sent on a monthly basis. The SMS text messages included explanatory text and a contact telephone number for the rheumatology team, and the introductory text on the electronic CHAQ assessments included a link to an information page on the Alder Hey website. Fig. 1 illustrates these aspects of the system.

### CHAQ completion rates

**Fig. 2:**
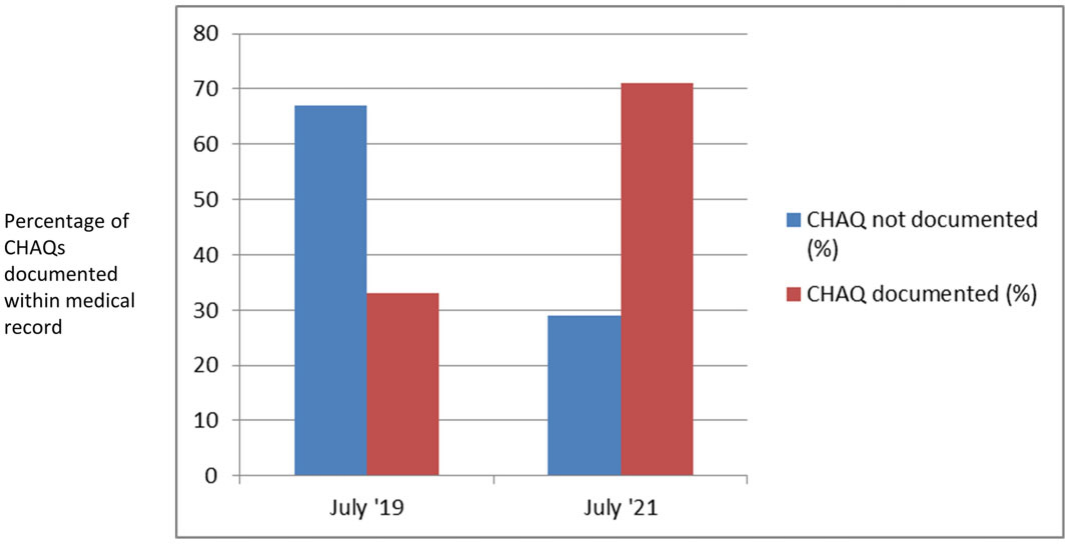
Bar chart demonstrating childhood HAQ completion rates before and after the introduction of the electronic patient-reported outcome measures system CHAQ: childhood HAQ.

Use of the ePROM system was associated with a statistically significant increase in the CHAQ completion rate. In the period before the implementation of the system, 33 of 100 (33%) assessed records included documentation of a CHAQ score; this increased to 47 of 65 (72%) after the introduction of the ePROM system (χ^2^ = 11.51; *P* < 0.05; see Fig. 2).

### Acceptability of the ePROM system

#### Acceptability to patients and their carers

The patient and parent ePROM questionnaire was completed by 24 respondents (no formal record of how many families declined to complete the questionnaire was captured). Respondents indicated positive baseline attitudes towards using health technologies [median response to ‘happy to use new tech for child’s health care’ question = 5 (strongly agree), interquartile range (IQR) = 0] and that the system was accessible to them [median responses to questionnaire items 2–4 = 5 (strongly agree), IQR = 0].

Respondents also indicated that they would find it acceptable to use the ePROM system again [median response to questionnaire item 6 = 5 (strongly agree), IQR = 0], and a majority reported preferring to complete ePROMs more frequently [*n* = 19/24 (79.2%)]. Respondents also perceived that the system was useful for their child’s care [median response to questionnaire item 7 = 5 (strongly agree), IQR = 0). The full responses to the questionnaire are summarized in Supplementary Table S2, available at *Rheumatology Advances in Practice* online.

#### Acceptability to clinical staff: results of focus group discussion

A focus group was conducted with 10 rheumatology HCPs, including consultant physicians, nurse specialists and occupational therapists, on 6 July 2021. The key themes identified from the discussion included positive feedback from families, more time for discussion during the consultation and an improved quality of clinical data. Focus group participants and themes can be found in Supplementary Tables S3 and S4, available at *Rheumatology Advances in Practice* online.

Negative aspects of using ePROMs included extra telephone calls to the administrative team for clarification about wording used in the CHAQ questionnaire and concerns that families with more limited access to financial resources might experience difficulties with accessing the system. The key themes identified in the focus group are outlined in Table 1.

**Table 1:** Summary of themes identified from health-care professional focus group discussion

Theme identified from analysis of focus group discussion	Illustrative quotes
Generally positive feedback	I really like it
	I find it really helpful
	I think this is working really, really well
Time saving	We don’t have to spend any time calculating the CHAQ score
Improved data quality	It’s fantastic [that CHAQ data] are captured within reports on Meditech [the hospital EPR]
	It gives the physiotherapists a baseline that they can work to and then they can repeat the CHAQ; I’ve found that incredibly helpful to get a sense of where the patient’s at
	I’m extremely excited about the fact that it [the CHAQ data] contributes to a set of core JIA criteria [data]
	I know in my own practice I haven’t been as robust as others about documenting and collecting the CHAQ when I’ve had it on paper, so I think my completion rate for the JIA core set [of data], you know, it’s going to improve significantly because of this
	That it’s not just a number anymore, and it pulls into the core set [JIA cores set of data] is fantastic
Access to CHAQ data ahead of clinic consultation	I found it helps to inform my clinical consultation both in terms of the report and in terms of the score and especially if I’ve seen that in advance of the patient coming in
	Being able to see the CHAQ before clinic and realize where there’s issues
Concerns and queries relating to when the ePROM messages are sent to families and carers	The patients who are in [to clinic] first thing … who are getting the CHAQ at half past eight [on the morning of the clinic appointment] are finding it more difficult [to complete the CHAQ before the appointment]
	Just in terms of patients ringing me, I’ve had a few different scenarios of wanting to know if they can complete it over the weekend if their appointment is on the Monday, will you get it back in time?
Concerns about the digital divide or equal access to digital systems	Are there any protections in place to ensure that some families have not been excluded or discriminated against and the potential bias that this could create if you only get the more kind-of well-off families being able to complete these questionnaires and are those questionnaires then going to feed back into data that we’re going to analyse?
	… [the] digital divide and inequality could be a real factor
	Families are offered paper copies [at the moment], but the more we use electronic systems the less families may be offered paper versions

CHAQ: childhood HAQ; EPR: electronic patient record; ePROM: electronic patient-reported outcome measures.

#### Acceptability to HCPs: results of technology acceptance model 2 (TAM2) survey

HCPs (*n* = 7; respondents included consultants, nurse specialists and occupational therapists) scored the ePROM highly across all TAM2 domains, including ‘Perceived Usefulness’ [median response = 7 (strong agreement); IQR = 0.25], ‘Output Quality’ [median response = 7 (strong agreement); IQR = 1] and ‘Perceived ease of use’ [median response = 7, IQR = 0]. The full responses to the questionnaire are summarized in Supplementary Table S5, available at *Rheumatology Advances in Practice* online.

## Discussion

This evaluation demonstrates the feasibility of integrating an automated ePROM system within an NHS EPR. The ePROM system was associated with an improvement in data quality and was highly acceptable to patients and HCPs. The system successfully resolved previous issues associated with the use of paper-based PROMs that included time-consuming completion and scoring processes and the risks of transcription and misfiling errors. The system also removed described barriers to the completion of PROMs away from hospital settings. High levels of automation and integration with existing health information technology systems were contextual factors that might have contributed to these successes. Potential unintended consequences of using the system included hypothetical concerns about the risk of families being excluded from using digital systems owing to resource constraints.

These findings suggest that ePROMs might help clinical teams to gain improved insights into the health status of their patients. Improvements in data quality might also help to improve audit and commissioning processes and might enable the integration of research into routine care, through the use of standardized, core data sets than can be collected routinely in clinical settings [18].

Strengths of this evaluation include the use of mixed methods to identify contextual factors that might have contributed to the positive findings described in the report and the large effect size in relationship to the change in PROM completion rates. Limitations include its single-centre design, the use of observational methods and the use of an unvalidated patient and parent ePROM questionnaire for testing the acceptability of the system, which might have been completed by a non-representative sample of families.

Future research approaches could therefore include evaluating the ePROM technology in additional settings; either using the CHAQ in an alternative centre or by using alternative questionnaires to assess additional PROM or patient-reported experience measures. Additional opportunities could include consideration of whether ePROM data might contribute to decisions about how frequently to arrange follow-up appointments for individuals.

### Conclusion

This evaluation confirmed the technical feasibility of integrating an electronic PROM directly into an NHS EPR system. Introduction of the ePROM was associated with improved data quality and was highly acceptable to patients and HCPs.

## Supplementary Material

rkac065_Supplementary_Data

## Data Availability

Data are available upon reasonable request by any qualified researchers who engage in rigorous, independent scientific research, and will be provided following review and approval of a research proposal and Statistical Analysis Plan (SAP) and execution of a Data Sharing Agreement (DSA). All data relevant to the study are included in the article.
