# Supplementary material for: Successful integration of an automated patient-reported outcome measure within a hospital electronic patient record
Source: Rheumatol Adv Pract. 2022 Aug 17;6(3):rkac065. doi: 10.1093/rap/rkac065 (PMC9447377; doi:10.1093/rap/rkac065)
Supplement: rkac065_Supplementary_Data [file rkac065_supplementary_data.doc]

# Successful integration of an automated Patient Reported Outcome Measure within a hospital electronic patient record – Supplementary Data

Matthew T. Neame^1,2^ , David Reilly^1^, Ajmal Puthiyaveetil^3^ , Liza McCann^3^, Kamran Mahmood^3^ , Beverley Almeida^3^ , Clare E. Pain^,2,3^ , Victoria Furfie^1^ , Andrew G. Cleary^3^*

Affiliations

1. Department of Information Technology, Alder Hey Children's Hospital, Liverpool, UK
2. Department of Women’s and Children’s Health, University of Liverpool, , Liverpool, UK
3. Department Rheumatology, Alder Hey Children's Hospital, Liverpool, UK

## Supplementary Table S1 – Patient and parent ePROM Questionnaire


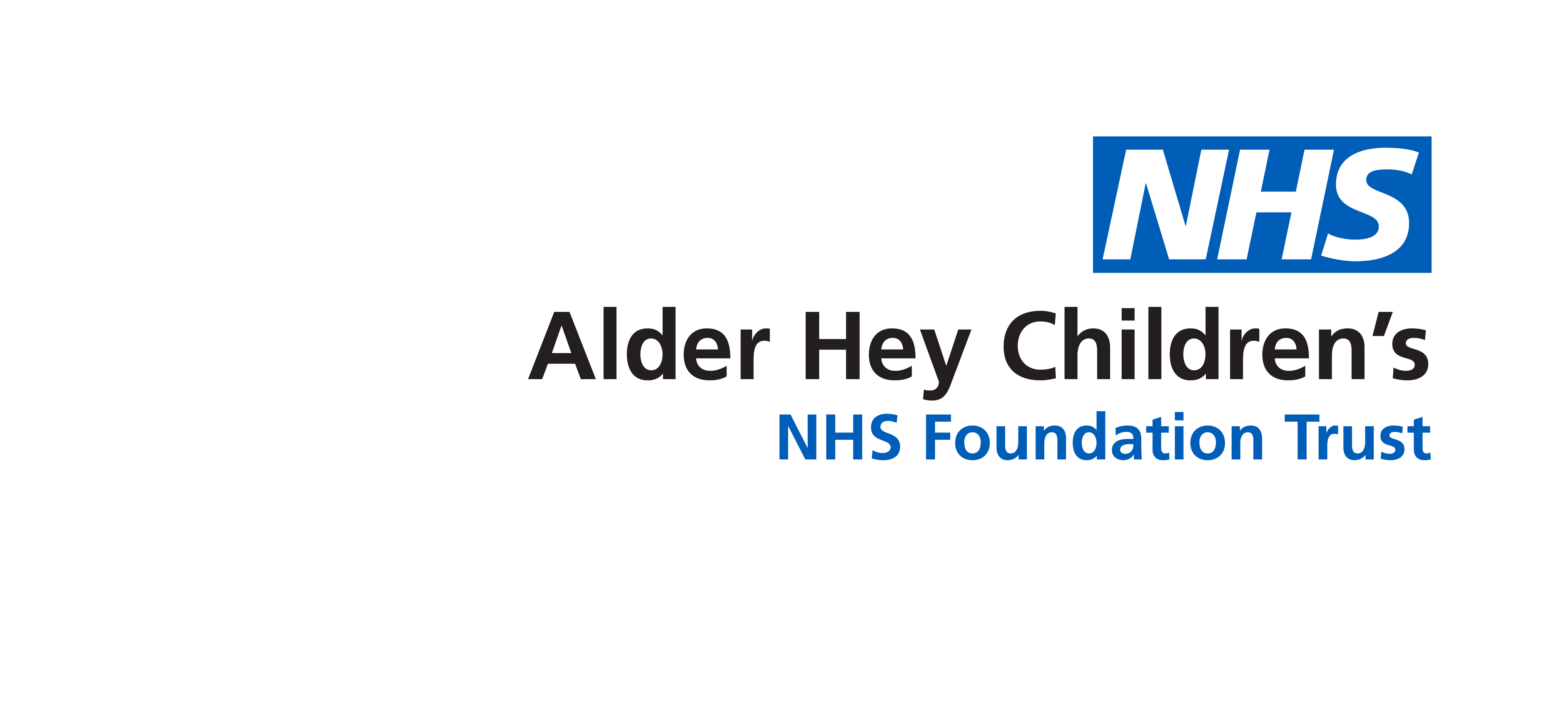


# Patient and parent ePROM questionnaire

**I have completed the ePROM at home before attending clinic Yes/No**

|  | Strongly  Disagree | Disagree | Neither agree or disagree | Agree | Strongly agree |
| --- | --- | --- | --- | --- | --- |
| In general, I am happy to use new technologies (e.g. the ePROM) as part of my/my child’s healthcare |  |  |  |  |  |
| I had no problem accessing the ePROM from my text message |  |  |  |  |  |
| The instructions were easy to understand |  |  |  |  |  |
| The ePROM was easy to complete |  |  |  |  |  |
| The language used in the ePROM was easy to understand |  |  |  |  |  |
| I would be happy to complete the ePROM again |  |  |  |  |  |
| I understand how the ePROM will be used to help my child’s healthcare |  |  |  |  |  |

**Please rank your top three within the scenarios below (1= fa**vourite)

| **1. I would prefer to complete health questionnaires…** | |
| --- | --- |
|  | A**t home** before attending clinic as an ePROM that is available to the doctor and nurse at my appointment |
|  | A **paper** version of the health questionnaire in the **hospital waiting room** |
|  | An **electronic** version of the health questionnaire in the **hospital waiting room** |
|  | A **paper** version of the questionnaire in a **room with a member of the clinical team** |
|  | An **electronic** version of the health questionnaire in a room **with a member of the clinical team** |

***Re*** ePROM please tick your preference below

| **Would you like a reminder regarding completion of the form?** | | | | | | | | | |
| --- | --- | --- | --- | --- | --- | --- | --- | --- | --- |
|  | Yes by email |  | Yes by text |  | | No | | |  |
| **When would you like the clinical team to request the ePROM?** | | | | | | | | | |
|  | Day before clinic appointment |  | Week before clinic appointment | |  | Two weeks before clinic appointment |  | Other  __________________ | |

| **3. Would you like the option to complete the ePROM more often, in between appointments?** | | |
| --- | --- | --- |
| Yes  If yes, how often____________________ | Yes, if I was feeling unwell | No |

| **4. Would you find it useful to complete the ePROM prior to a telephone conversation with the nurses?** | |
| --- | --- |
| Yes | No |

| **5. Who do you think should complete the ePROM?** | | | |
| --- | --- | --- | --- |
| **If patient aged under 11** | | | |
| Parent/carer | Parent/carer and patient | Other  __________________ | |
| **If patient aged over 11** | | | |
| Parent/carer | Patient | Both patient and parent/carer to complete own version of form | Other  __________________ |

**Are you aware of any unwanted, unhelpful or harmful effects that may have been caused by using the ePROM? *Please comment below***

**Any final** comments or suggestions

|  |
| --- |

## Supplementary Figure S1 – Introductory slides used during focus group conducted with health care professionals


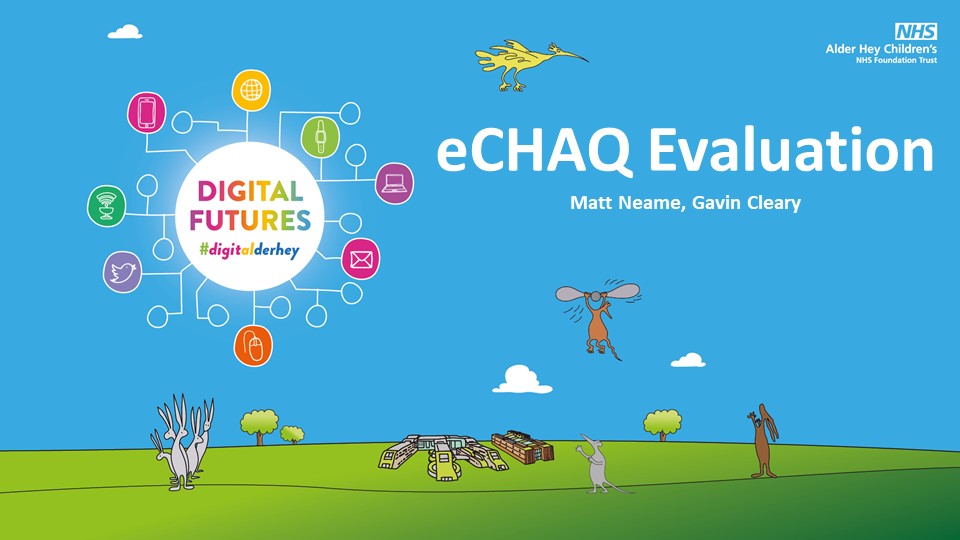


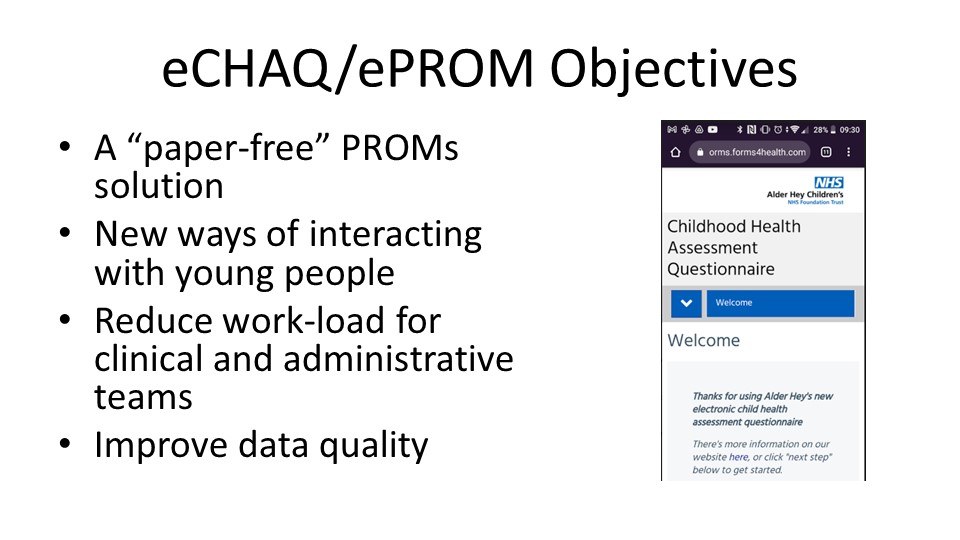


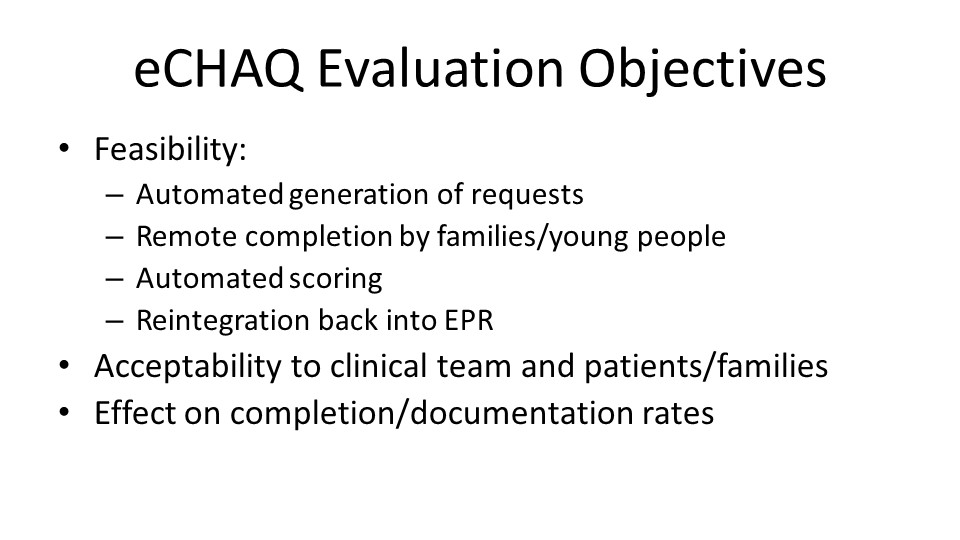


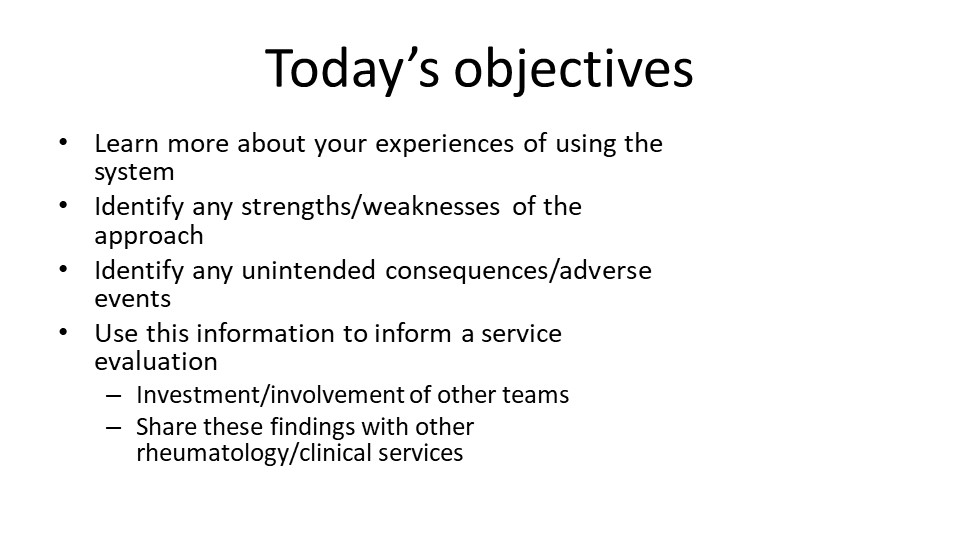


## Supplementary Figure S2 – Screenshot of patient information page hosted on Alder Hey Children’s Hospital NHS Trust website


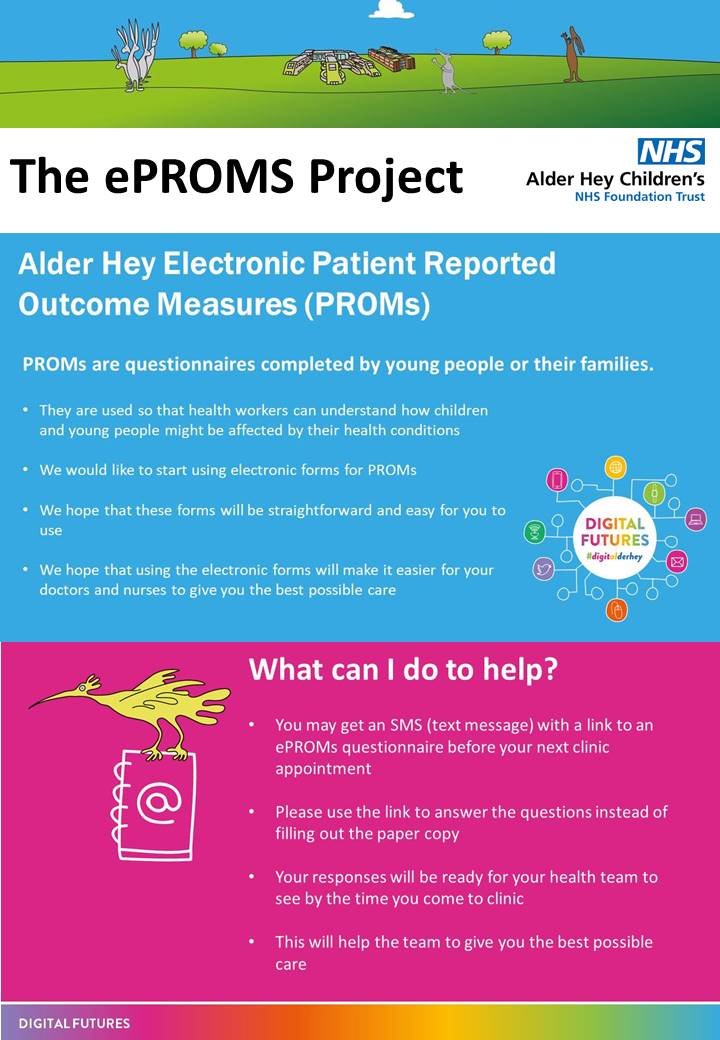


## Supplementary Table S2 – Summary of responses to Patient and Parent ePROM questionnaire

| **Questionnaire Item** | **Median Score on Likert Scale (1 = Strongly Disagree, 5 = Strongly Agree) (Interquartile Range) (n = 24)** |
| --- | --- |
| In general, I am happy to use new technologies (e.g. the ePROM) as part of my/my child’s healthcare | 5 (0) |
| I had no problem accessing the ePROM from my text message | 5 (0) |
| The instructions were easy to understand | 5 (0) |
| The ePROM was easy to complete | 5 (0) |
| The language used in the ePROM was easy to understand | 5 (0) |
| I would be happy to complete the ePROM again | 5 (0) |
| I understand how the ePROM will be used to help my child’s healthcare | 5 (0) |
| **I would prefer to complete health questionnaires…** | |
| A**t home** before attending clinic as an ePROM that is available to the doctor and nurse at my appointment | 23/24 (95.8%) |
| A **paper** version of the health questionnaire in the **hospital waiting room** | 0/24 (0.0%) |
| An **electronic** version of the health questionnaire in the **hospital waiting room** | 1/24 (4.2%) |
| A **paper** version of the questionnaire in a **room with a member of the clinical team** | 0/24 (0.0%) |
| An **electronic** version of the health questionnaire in a room **with a member of the clinical team** | 0/24 (0.0%) |
| **Would you like a reminder regarding completion of the form? (more than one response possible)** | |
| Yes, by email | 4/24 (16.7%) |
| Yes, by text (SMS) | 21/24 (87.5%) |
| No | 0/24 (0.0%) |
| **When would you like the clinical team to request the ePROM?** | |
| Day before clinic appointment | 8/24 (33.3%) |
| Week before clinic appointment | 15/24 (62.5%) |
| Two weeks before clinic appointment | 1/24 (4.2%) |
| **Would you like the option to complete the ePROM more often, in between appointments?** | |
| Yes | 12/24 (50%) |
| Yes, if I was feeling unwell | 7 (29.2%) |
| No | 5 (20.8%) |
| **Would you find it useful to complete the ePROM prior to a telephone conversation with the nurses?** | |
| Yes | 20 (83.3%) |
| No | 4 (16.7%) |

## Supplementary Table S3 – Focus group participants by professional group

| **Professional group** | **Number of participants** |
| --- | --- |
| Consultant (attending) paediatric rheumatologist | 2 |
| Paediatric rheumatology nurse specialist | 2 |
| Occupational Therapist | 2 |
| Administrative support staff | 1 |
| Clinical psychologist | 1 |
| Pharmacist | 1 |
| Training grade doctor | 1 |

## Supplementary Table S4 – Summarised themes identified from focus group discussion

| **Theme identified from analysis of focus group discussion** | **Illustrative quotes** |
| --- | --- |
| Generally positive feedback | *I really like it* |
|  | *I find it really helpful* |
|  | *I think this is working really, really well* |
| Time saving | *We don’t have to spend any time calculating the CHAQ score* |
| Improved data quality | *It’s fantastic [that CHAQ data] is captured within reports on Meditech [the hospital EPR]* |
|  | *It gives the physiotherapists a baseline that they can work to and then they can repeat the CHAQ - I've found that incredibly helpful to get a sense of where the patient’s at* |
|  | *I’m extremely excited about the fact that it [the CHAQ data] contributes to a set of core JIA criteria [data]* |
|  | *I know in my own practice I haven’t been as robust as others about documenting and collecting the CHAQ when I’ve had it on paper so I think my completion rate for the JIA core set [of data], you know, it’s going to improve significantly because of this* |
|  | *That it’s not just a number anymore, and the it pulls into the core set [JIA cores set of data] is fantastic* |
| Access to CHAQ data ahead of clinic consultation | *I found it helps to inform my clinical consultation both in terms of the report and in terms of the score and especially if I’ve seen that in advance of the patient coming in* |
|  | *Being able to see the CHAQ before clinic and realise where there’s issues* |
| Concerns and queries relating to when the ePROM messages are sent to families and carers | *The patients who are in[to clinic] first thing...who are getting the CHAQ at half past eight [on the morning of the clinic appointment] are finding it more difficult [to complete the CHAQ before the appointment]* |
|  | *Just in terms of patients ringing me I’ve had a few different scenarios of wanting to know if they can complete it over the weekend if their appointments on the Monday will you get it back in time.* |
| Concerns about the “digital divide” or equal access to digital systems | *Are there any protections in place to ensure that some families have not been excluded or discriminated against and the potential bias that this could create if you only get the more kind of well-off families being able to complete these questionnaires and are those questionnaires then going to feed back into data that we’re going to analyse* |
|  | *[the] digital divide and inequality could be a real factor* |
|  | *Families are offered paper copies [at the moment] but the more we use electronic systems the less families may be offered paper versions* |

## Supplementary Table S5 - Summarised results from TAM2 questionnaire

| **Questionnaire Item** | **Median Score on Likert Scale (1 = Strongly Disagree, 7 = Strongly Agree) (Interquartile Range) (n = 7)** |
| --- | --- |
| *Intention to use* | |
| Assuming I have access to the system, I intend to use it | 7 (0) |
| Given that I have access to the system, I predict that I would use it | 7 (0) |
| *Perceived usefulness* | |
| Using the system improves my performance in my job | 7 (0.5) |
| Using the system in my job improves my productivity | 7 (0.5) |
| Using the system enhances my effectiveness in my job | 7 (0.5) |
| I find the system to be useful in my job | 7 (0) |
| *Perceived ease of use* | |
| My interaction with the system is clear and understandable | 7 (0) |
| Interacting with the system does not require a lot of my mental effort | 7 (1) |
| I find the system to be easy to use | 7 (0) |
| I find it easy to get the system to do what I want it to do | 7 (0.5) |
| *Subjective norm* | |
| People who are important to me think that I should use the system | 5 (2.75) |
| People who influence my behaviour think that I should use the system | (3) |
| *Voluntariness* | |
| My use of the system is voluntary | 7 (1.5) |
| My supervisor does not require me to use the system | 2 (4.75) |
| Although it might be helpful, use of the system is certainly not compulsory in my job | 4 (2.75) |
| *Image* | |
| People in my organisation who use the system have more prestige than those who do not | 2 (2.25) |
| People in my organisation who use the system have a high profile | 3.5 (1.75) |
| Having the system is a status symbol in my organisation | 4 (0.25) |
| *Job relevance* | |
| In my job, usage of the system is important | 7 (0) |
| In my job, usage of the system is relevant | 7 (0) |
| *Output quality* | |
| The quality of the output I get from the system is high | 7 (0.5) |
| I have no problem with the quality of the system's output | 7 (0.75) |
| *Result demonstrability* | |
| I have no difficulty telling others about the results of using the system | 7 (0.75) |
| I believe I could communicate to others the consequences of using the system | 7 (0.75) |
| The results of using the system are apparent to me | 7 (0.75) |
| I would have difficulty explaining why using the system may or may not be beneficial | 1 (1.5) |

## Supplementary Data S1. Evaluation protocol

**Evaluation of the feasibility and acceptability of an electronic Patient Reported Outcome Measurement system in a paediatric rheumatology clinic – Evaluation protocol**

Dr Matthew Neame, Paediatric ST7

Dr Gavin Cleary, Consultant Paediatric Rheumatologist

**BACKGROUND**

Patient Reported Outcome Measures (PROMs) are tools for assessing health conditions from a patient or caregiver’s perspective. PROMs are most frequently designed as standardised, validated questionnaires that can be used to measure the health effects (outcomes) that are of most importance to patients and their families; these might include levels of physical or social functioning, severity of symptoms, or general wellbeing.^1^

PROMs can be used to measure these effects in research settings or as part of routine clinical care. When they are used to measure the effects of interventions administered during research, PROMs can help to identify the treatments with the most beneficial effects. When PROMs are used as part of routine clinical care, they may help healthcare workers and patients to track the progress of their treatments, and may help to enable objective comparisons of the services provided at different healthcare organisations^1^.

The Child Health Assessment Questionnaire (CHAQ) is a PROM that has been validated for use in Juvenile Idiopathic Arthritis – a rheumatological condition that causes pain and inflammation in the joints of children and young people^2,3^. PROMs like the CHAQ have an established role in rheumatology research and are included in the standardised outcome sets that are used to measure the effects of new treatments^4^. In routine clinical care, PROMs have been used for monitoring symptom control and have been proposed as an important source of data for submission to rheumatology disease registries.^5^

However, despite the potential benefits of using PROMs, there are barriers that may prevent their use in clinical practice. These factors include:

1. Paper-based questionnaire PROMs usually require manual scoring – this can be time-consuming and may result in calculation errors
2. There may be logistical challenges associated with completing the PROMs away from clinical settings
3. PROM scores need to be manually transcribed into the computer systems that are used to maintain clinical records and disease registry databases – this can also be time-consuming and may result in transcription errors

Electronic PROM (ePROM) systems may help to overcome some of these barriers by: enabling remote completion of PROMs; enabling automated scoring of questionnaires; and negating the need to transcribe the calculated scores into clinical records or databases.

This protocol has therefore been developed with the aim of investigating the technical feasibility and acceptability of using an ePROM system configured for administration of CHAQs as part of a specialised children’s rheumatology service. Specific objectives will be to test the feasibility of developing and implementing an ePROM system that automatically generates requests to complete PROMs based on clinic attendance, and which automatically integrates the PROMs data back into the existing electronic systems used in clinical care. Further objectives will be to evaluate the fidelity of the intervention (the degree to which participants used it as intended) and its acceptability to patients, care-givers and clinical staff.

**METHODS**

**Study Design and Setting**

This evaluation will be conducted within the rheumatology department at Alder Hey Children’s hospital, a specialist children’s hospital in the United Kingdom.

Paper-based CHAQ assessments have been used as a part of routine clinical care within the department for several years. These questionnaires are provided to patients in the clinic waiting room and are then passed to the clinicians conducting clinic appointments for scoring. CHAQ scores are then manually transcribed into the hospital’s Electronic Health Record (EHR) (MEDITECH V6.08, Boston, USA) and the paper questionnaires are returned to the medical records department for scanning into an electronic document management system.

In order to address some of the challenges associated with using paper-based PROMs an ePROMs CHAQ system has been developed collaboratively by the Alder Hey Information technology team and an independent technology provider (AireLogic, Leeds, UK). The ePROMs CHAQ system has been piloted in the department from March 2021. In keeping with recommended approaches for evaluating Health Information Technologies we will use mixed methods (quantitative and qualitative) to conduct an evaluation of the ePROM CHAQ system’s acceptability, fidelity and feasibility as a technical solution over a three month period between June and August 2021.^6-8^

**Technical Feasibility Evaluation**

This evaluation will be conducted using a descriptive case study design. The key aspects of the ePROMs CHAQ system will be described in accordance with recommendations for reporting Health Information Technologies (HITs)^9^. The description will include overviews of:

1. The existing Health Information Technology (HIT) Infrastructure
2. Key technical features of the ePROMs system
3. Configurations made to the system during the evaluation period
4. The methods used to enable the integration and interoperation of the ePROMs systems with other elements of the hospital’s HIT infrastructure

The case study will include a review of whether the key technical aims of the ePROMs CHAQ system were achieved. These aims include:

1. Automated generation of Short Messenger Service (SMS) text messages to patients due to attend the rheumatology clinics at Alder Hey NHS Foundation Trust
2. The SMSs should include a unique URL enabling young people or their families to complete the CHAQ remotely via a mobile device
3. The completed CHAQs should be automatically transferred back to the patient’s account within the hospital’s EHR
4. The record in the EHR should include the calculated CHAQ score as well as the individual responses to items included in the CHAQ assessment

**Evaluation of Intervention Fidelity**

Intervention fidelity has been described as the “the extent to which an intervention was delivered as conceived and planned”. ^10^ Intervention fidelity will be evaluated by measuring the frequency with which the ePROM CHAQ system was successfully used in clinical practice. A before-after study design will also be used to compare the frequencies with which CHAQ scores were recorded in the clinical records of eligible children and young people before and after the introduction of the ePROM CHAQ system.

The planned use of the intervention is to enable each child or young person who is scheduled to attend a face-to-face, telephone or video clinic consultation provided by the Alder Hey rheumatology department to complete a CHAQ using the ePROM system. The intervention will therefore be defined as having been successfully delivered if, following an attendance at a clinic appointment, a child or young person’s clinical record includes a calculated CHAQ score *and* a copy of the completed ePROM CHAQ questionnaire.

These factors will be evaluated using a manual review of the clinical records of each child who attended a scheduled appointment with the rheumatology department over a two week period in July 2021. Eligible patients will be identified using the clinic codes which relate to scheduled rheumatology appointments. Intervention fidelity will be expressed as the percentage of the total number of eligible clinical records which included the calculated CHAQ score *and* a copy of the completed ePROM CHAQ questionnaire.

The evaluation will also include an assessment of the proportion of children and young people whose clinical records included a calculated CHAQ score before the introduction of the ePROMs CHAQ system. This value will be calculated by using the same methods to review the clinical records of children and young people who attended outpatient rheumatology clinic appointments over a two week period in July 2019.

The Chi-square test will be used to assess whether the introduction of the ePROM CHAQ system was associated with a statistically significant change in the frequency with which completed CHAQ scores were documented in the clinical records.

**Acceptability**

Acceptability to children, young people and their care-givers

The acceptability of the intervention to children, young people and their care-givers will be assessed by surveying individuals who are attending rheumatology clinics. The survey will be conducted using a semi-structured questionnaire (see appendix). The questionnaire has been developed by the Alder Hey rheumatology team.

The questionnaire will be provided to children and their caregivers in clinic waiting rooms in paper form, along with a participant information sheet explaining the purpose of the survey and reassuring families that participation is on a voluntary basis (see appendix). Paper-based questionnaires will be used in order to ensure that the views of families who may not have received or responded to electronic communication methods can also be identified.

The objective will be to collect questionnaire responses from at least 20 patients or caregivers attending at least five scheduled clinics over the evaluation period.

The results of the questionnaires will be transcribed into a spreadsheet for analysis. The questionnaire items that include Likert scale responses will be analysed using descriptive statistical methods. Thematic analysis methods will be used to assess the free text responses.

Acceptability to clinical staff

The acceptability of the ePROM CHAQ to clinical staff will be evaluated using a structured questionnaire and a focus group. Any members of the rheumatology team who conduct outpatient assessments will be eligible for inclusion in the evaluation of acceptability. The aim will be to collect questionnaire responses from at least 10 members of staff and to conduct focus group discussions with at least three members of medical staff and three nurse specialists.

The Technology Acceptance Model 2 (TAM2) questionnaire^11,12^ (see Appendix) has been validated for evaluating the acceptability of technologies introduced into workplace settings and will be used to survey clinical staff. The questionnaire measures key constructs that have been demonstrated to predict usage intentions and the acceptance of workplace information technology systems by employees^13^. The items are measured using a seven point Likert scale. The results of the survey will be analysed using descriptive statistical methods.

The focus group session will involve semi-structured discussions with staff members who have used the ePROM CHAQ system in clinical settings. Consenting participants will be invited to discuss key aspects of the system including:

- Usefulness
- Usability
- Suggestions for improving the system
- Any adverse events or unintended consequences of using the system

Discussion points will be contemporaneously by an evaluator during the session and thematic analysis methods will be used to identify key themes arising from these discussions.

**Ethics**

Use of the NHS Health Research Authority decision tool^14^ has identified that this proposal would constitute a service evaluation exercise which would not require approval from a Research Ethics Committee.

**DISCUSSION**

The use of electronic PROMs systems may help to promote the use of PROMs in routine clinical care. This evaluation has been designed to assess the technical feasibility and acceptability of introducing an automated ePROMs system into a rheumatology clinic in a specialist children’s hospital. The results of this evaluation may provide important insights into factors that affect the acceptability of this intervention to patients, caregivers and clinical staff. These findings may also provide insights into design features that can improve or diminish the acceptability of future iterations of the ePROMs system.

**REFERENCES**
